# Healing patterns of choroidal tubercles after antitubercular therapy: A photographic and OCT study

**DOI:** 10.1007/s12348-011-0040-z

**Published:** 2011-09-07

**Authors:** Salil Mehta

**Affiliations:** Department of Ophthalmology, Lilavati Hospital and Research Center, Bandra Reclamation, Mumbai, India

**Keywords:** Choroidal tubercles, Healing, Fundus photography, Optical coherence tomography

## Abstract

A 28-year-old female patient with disseminated tuberculosis and choroidal tubercles on a regimen of systemic antitubercular therapy underwent fundus photography and optical coherence tomography (OCT). This was carried out monthly until complete healing of the tubercle was seen. The tubercle consisted of a central white-yellow core, consistent with choroiditis, with a faint hyperpigmentation surrounding it. There was a surrounding diffuse rim of inflammation. By the second month, the hyperpigmented rim was more prominent as were the outer edges of both the central core and the outer rim. Over time, the outer rim had largely faded with concurrent scar formation in the core. The initial OCT analysis revealed a raised RPE-choriocapillaris complex. With healing, there was a marked reduction in the choroidal lesional height suggesting resolution.

## Introduction

Choroidal tubercles are a common manifestation of ocular tuberculosis and represent a direct mycobacterial infection of the choroid. Extensive data exists on their prevalence and significance but their healing following appropriate therapy has been sparsely documented. We report the photographic and optical coherence tomography (OCT) findings during a 4-month period in one patient.

## Case report

A 28-year-old female patient was referred to us for evaluation. She had a history of high-grade fever and severe headache in the preceding 2 months and had undergone a systemic evaluation during the preceding week. She was anemic (hemoglobin 10.3 g%) but had normal total and differential white cell counts. Her erythrocyte sedimentation rate (ESR) was 84 mm/h (normal <20 mm/h). Investigations for renal and liver functions were normal. Her serum angiotensin-converting enzyme (ACE) was within normal limits. Her mantoux test was strongly positive at 23 × 25 mm. Magnetic resonance imaging (MRI) of the brain showed multiple ring-shaped lesions in the cerebral and cerebellar areas, with perilesional edema and contrast enhancement. Computed tomography (CT) of the chest revealed multiple discrete nodular opacities in the lung parenchyma bilaterally and enlargement of the mediastinal nodes. A diagnosis of disseminated tuberculosis had been made, and she had been started on a four-drug antitubercular drug regimen (initial 2 months of ethambutol [15 mg/kg body weight], rifampicin [10 mg/kg], isoniazid [5 mg/kg] and pyrazinamide [25 mg/kg] followed by 7 months of rifampicin and isoniazid).

On examination, her visual acuity was 6/6 bilaterally. A slit lamp examination was normal in either eye. Dilated fundus examination was normal in the right eye and showed two white to yellow 1/3 to 1/2 disc diameter sized choroidal lesions (in the nasal quadrant and temporal to the macula) consistent with tubercles. She underwent fundus photography and OCT (Stratus, Carl Zeiss Meditec, Oberkochen, Germany). We used the line scan program (normalize + align analysis) on a monthly basis for four visits, until clinical healing of the tubercles was achieved.

**Fig. 1:**
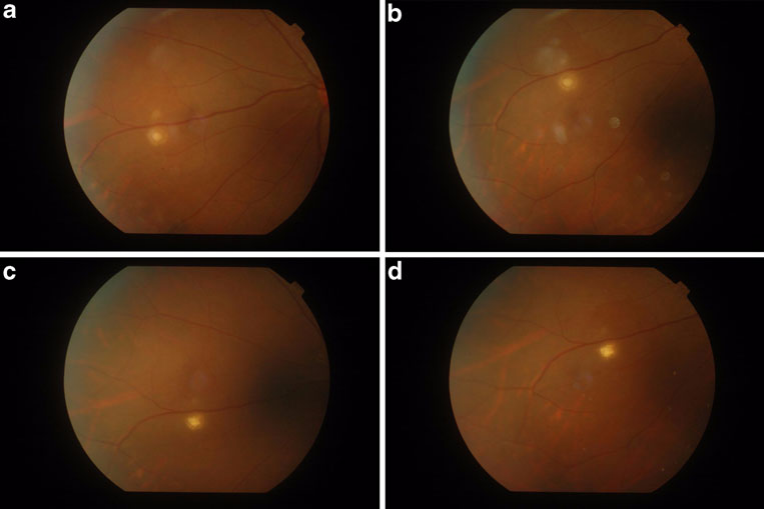
Montage of fundus photographs showing the clinical healing of tubercles from initial visit (central core of choroiditis with surrounding inflammatory rim) to month four (scar formation)

**Fig. 2:**
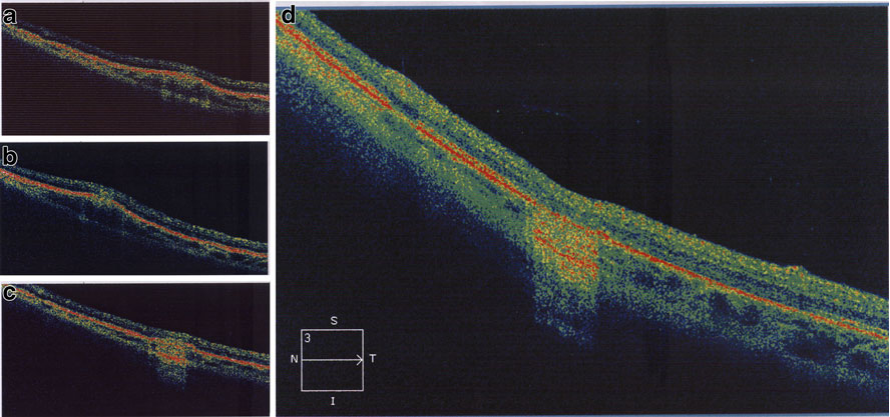
Montage of OCT images showing the tomographic changes in a choroidal tubercle from diagnosis (raised RPE-choriocapillaris complex) to resolution (flattened scar with shadowing)

The tubercle was approximately 1/2 DD in size and consisted of a central white-yellow core, consistent with choroiditis, with a faint hyperpigmentation surrounding it. There was a surrounding diffuse rim of inflammation (Fig. 1a), consistent with a surrounding retinal inflammation. By the second month, the hyperpigmented rim was more prominent, and the outer edges of both the central core and the outer rim were more distinct (Fig. 1b). By the third month, the outer rim had largely faded with concurrent scar formation in the core (Fig. 1c). This scar (referred to as a “parchment” scar in earlier publications) was complete by the fourth month (Fig. 1d). At the initial visit, OCT analysis revealed a raised RPE-choriocapillaris complex consistent with a raised choroidal lesion at the initial visit. The overlying retina appeared normal (Fig. 2a). These findings persisted into the second month (Fig. 2b). At the third month, there was a marked reduction in the choroidal lesional height suggesting resolution (Fig. 2c). At the fourth month, there was a complete flattening of the choroidal lesion with a hyper reflectivity and “shadowing” of the area of the lesion. The overlying retina appeared slightly depressed, suggesting a dense contracted scar formation (Fig. 2d).

## Discussion

Published literature has described the healing of tubercles as either a process of complete resolution or alternatively scar formation [1, 2]. The latter process consists of increasingly distinct borders with increasing marginal pigmentation and resulting in the formation of a white scar. This was the sequence of events we observed. The gradual healing of the infection and subsequent reduction of edema and inflammation was manifested clinically by the reduction and eventual disappearance of the surrounding inflammation and the appearance of pigmentation and scar formation. This correlated with the OCT findings of a raised choroidal lesion that resolved with scar formation. The healing was complete by the fourth month in our patient but this response may vary due to factors such as bacterial virulence, host immunity and drug efficacy issues.
